# A Novel Prognostic Index Based on Alternative Splicing in Papillary Renal Cell Carcinoma

**DOI:** 10.3389/fgene.2019.01333

**Published:** 2020-01-29

**Authors:** Zhipeng Wu, Jinhui Liu, Rui Sun, Dongming Chen, Kai Wang, Changchun Cao, Xianlin Xu

**Affiliations:** ^1^Department of Urology, Sir Run Run Hospital, Nanjing Medical University, Nanjing, China; ^2^Department of Gynecology, The First Affiliated Hospital of Nanjing Medical University, Nanjing, China

**Keywords:** alternative splicing, prognostic index, papillary renal cell carcinoma, splicing factor, The Cancer Genome Atlas

## Abstract

**Background:**

Papillary renal cell carcinoma (pRCC) is a heterogeneous multifocal or isolated tumor with an invasive phenotype. Previous studies presented that alternative splicing, as a crucial posttranscriptional regulator in gene expression, is associated with tumorigenesis. However, the association between alternative splicing and pRCC has not been clarified

**Methods:**

The RNA sequencing data and clinical information were downloaded from The Cancer Genome Atlas database and mRNA splicing profiles from TCGASpliceSeq. The percent spliced in data of alternative splicing merged with survival information was firstly calculated by univariate Cox regression analysis to screen for survival‐associated alternative splicing events, and survival‐associated alternative splicing events were then analyzed by Gene Ontology categories using Kyoto Encyclopedia of Genes and Genomes. Meanwhile, the least absolute shrinkage and selection operator Cox analysis and multivariate Cox analysis were performed to calculate the prognostic index for each alternative splicing type. In addition, clinical factors were introduced to assess the performance of prognostic index.

**Results:**

A total of 4,084 candidate survival-associated alternative splicing events in 2,558 genes were screened out. Patients were divided into the low-risk group and the high-risk group based on the median prognostic index value. The Kaplan-Meier survival analysis (p < 0.05) and receiver operating characteristics curves (AUC>0.9) indicated that prognostic index was effective and stable for predicting the prognosis of pRCC patients. Furthermore, a regulatory network was constructed incorporating alternative splicing events and survival-associated splicing factors.

**Conclusion:**

Our study provides new insights into the mechanism of alternative splicing events in tumorigenesis and their clinical potential for pRCC.

## Introduction

Papillary renal cell carcinoma (pRCC), which accounts for up to 15% of renal cell carcinoma, is the second most common histological subtype of kidney cancer (Jonasch et al., 2014; Malouf et al., 2016). PRCC emerges as either indolent localized tumor or aggressive metastatic cancer (Delahunt and Eble, 1997), but the biological basis for this difference remains unidentified. Vascular endothelial growth factor (VEGF) pathway has been proven to be involved in metastatic pRCC (Armstrong et al., 2016), but we still speculate that multiple mechanisms lie behind these pRCC with diverse presentations. Thus, we designed this systematic and comprehensive analysis to drill into the oncogenic mechanism of pRCC.

High-throughput sequencing has revolutionized human genomics and the research in this field. The current number of human genes is still controversial. Up to now, people’s statistics on the number of genes are constantly changing (Pertea and Salzberg, 2010; Pertea et al., 2018). The GENCODE (Frankish et al., 2019) genome maintained by EBI currently counts 19,965 protein-coding genes, 17,910 long noncoding RNA genes, and 7,576 small noncoding genes in human (https://www.gencodegenes.org/human/stats.html). The database RefSeq (O'Leary et al., 2016), managed by the National Center for Biotechnology Information, lists 20,203 protein-coding genes and 17,871 noncoding genes. Regardless of the specific number of genes, given the limited number of human genes, alternative splicing (AS) serves as a key mechanism producing myriads of proteins (Tang et al., 2013; Bowler et al., 2018). AS is regulated by spliceosome, a large and highly dynamic protein complex constructed by nearly 200 protein components and five small nuclear ribonucleic acids (Agrawal et al., 2018). Dysregulation of splicing factors (SFs) can distort mRNA splicing programs, which could result in cancer development and progression (Grosso et al., 2008). Studies have also shown that aberrant AS events during transcription, which are tissue-specific and stage-specific, can evoke tumorigenesis (Wang et al., 2008; Kahles et al., 2018; Wan et al., 2019).

In this study, to clarify the AS events and its clinical implications in pRCC, AS events and complete clinical information from the TCGA database were analyzed. A prognostic model was formed to predict the prognosis of pRCC according to the survival information. Meanwhile, a regulatory network was constructed to evaluate the correlation between AS events and SFs, and identify several key factors which might exert important functions in occurrence and development of pRCC.

## Materials and Methods

### Data Collection of AS Events

**Figure 1:**
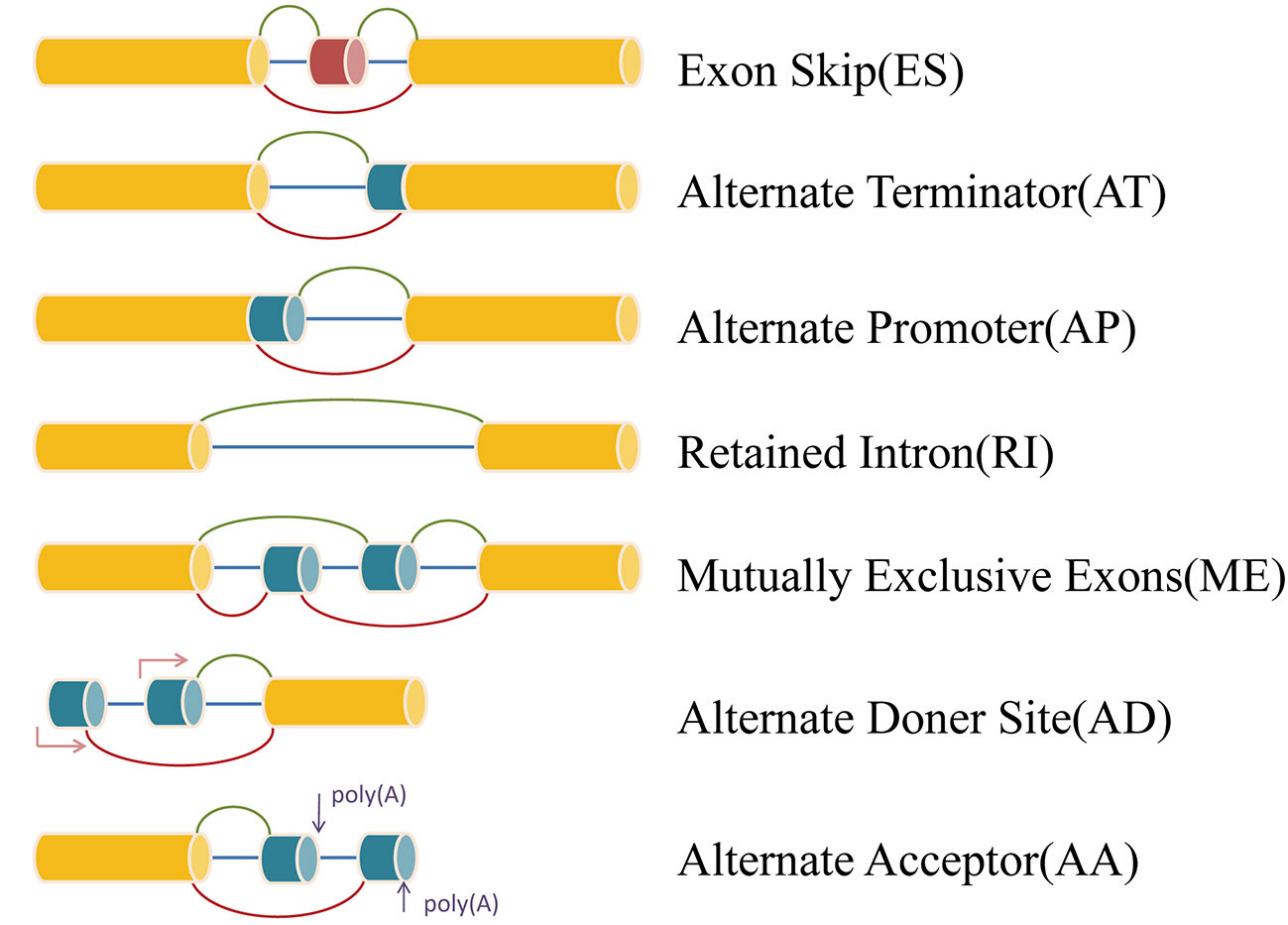
Representative model of seven types of alternative splicing.

RNA sequencing data (level 3) and clinical information of The Cancer Genome Atlas (TCGA) KIRP cohorts were obtained from the TCGA data portal (https://portal.gdc.cancer.gov/). Analysis of mRNA splicing profiles in pRCC was conducted with the aid of SpliceSeq (Ryan et al., 2016), java that explicitly quantifies RNA-Seq reads and identifies its possible functional changes as a consequence of AS in the context of transcript splice graphs. AS events were divided into seven types including exon skip (ES), mutually exclusive exons (ME), retained intron (RI), alternate promoter (AP), alternate terminator (AT), alternate donor site (AD), and alternate acceptor site (AA) (**Figure 1**). Meanwhile, we downloaded the Percent Spliced In (PSI) value (>75%) for pRCC patients. The PSI value, ranging from zero to one, was used in quantifying AS events.

### Identification of Survival-Associated AS Events

A total of 32 adjacent normal tissues and 289 pRCC tissues were collected from TCGA. The number of AS events and genes involved was showed by UpSet plot using “UpSetR” package in R (Conway et al., 2017). Univariate Cox regression analysis was used to screen out the candidate AS events (P < 0.05).

### Functional Annotation

The parent genes of survival‐associated AS events were subjected to functional enrichment analyses. Gene ontology (GO) term enrichment analysis and Kyoto Encyclopedia of Genes and Genomes (KEGG) pathway analysis were performed using “clusterProfiler” package in R (Yu et al., 2012). A p-value and q-value both smaller than 0.05 in GO and KEGG was considered significant.

### Survival Analysis

The result of univariate Cox regression analysis in identifying survival-associated AS events was shown by Volcano plot using “ggplot2” in R (**Figure 4A**). Seven types of AS events were revealed by Bubble chart respectively. Each chart contained the top 20 significant survival-associated AS events of corresponding types. LASSO method was then employed for the regression of high-dimensional predictors. LASSO Cox regression model was used to determine the ideal coefficient for each prognostic feature and to estimate the deviance likelihood via 1-standard error (SE) criteria. The coefficients and partial likelihood deviance were calculated with the “glmnet” package in R (Friedman et al., 2010).

### Construction and Validation of a Prognostic Model

The result of LASSO Cox regression was then submitted to multivariate Cox analysis to evaluate the independent prognostic value of each gene and construct an independent prognosis model.

The risk score model for prediction based on survival-associated AS events were calculated by multiplying the PSI values of prognostic indictors and the regression coefficient calculated by the multivariate Cox regression analysis.

$$\text{Risk Score based on eventes(patient)}=\sum_{i}\text{Coefficient}\left({\text{mRNA}}_{i}\right)\times \text{PSI value}\left({\text{mRNA}}_{i}\right)$$

The specific calculation formula was shown in **Table 1**. Patients were then divided into two groups based on the median levels of risk score. This prognostic model and patient survival information were merged. Kaplan-Meier survival curves were conducted to identify the prognostic ability of prediction models. Area under the curve (AUC) value for the ROC curves of each prognostic model was calculated by survivalROC package in R. Besides, univariate and multivariate analysis were performed containing the risk score of prognostic models and important clinical features for pRCC. Patients with incomplete clinical information and less than 90 days of OS were excluded, and only 129 samples were deemed qualified. Finally, a nomogram was constructed using the “rms” package on R. The calibration of this nomogram was assessed by calibration curves.

**Table 1:** Prognostic signatures for papillary renal cell carcinoma.

Type	Formula	AUC
AA	FKBP8|48446|AA*−8.261326216+ITGB1BP1|52617|AA*−3.645087449+FAM213A|12365|AA*−3.591154025+MYL6|22381|AA*−16.63167012+PILRB|80930|AA*4.461677112+TRPT1|16579|AA*−28.05557115+RPS24|12297|AA*−2.123512398+ENTPD6|58863|AA*−4.538790703+SPATA20|42429|AA3.809741969	0.967
AD	TCEB1|84216|AD*−3.227392376+ATP5J|60266|AD*−6.409262477+IRF3|51027|AD*−5.374919151+CFL2|27169|AD*−4.984160824+ATP6V1H|83836|AD*−14.79100606+AKT1S1|51111|AD*−6.962315249+PQBP1|89029|AD*−2.068032636	0.861
AP	UNG|24277|AP*4.194935004+NHS|88586|AP*−2.822266615+DYNC1I2|55939|AP*−6.138633492+LIMA1|21688|AP*3.922489143+PMEPA1|59946|AP*1.640017308+RNF220|2555|AP*−4.636337467	0.961
AT	CLDN11|67616|AT*1.439647629+SLC25A48|73462|AT*-3.684109126+KIF4A|89373|AT*2.503402505+RBM39|59235|AT*−22.33853542+BCAM|50347|AT*3.282115813+LARP1B|70567|AT* −3.356001505	0.959
ES	YIF1A|17008|ES*−20.56958015+RPS24|12295|ES*−1.999160275+C16orf13|265882|ES*−2.503302918+ARHGEF10|82562|ES*−2.479391656+PAX8|55049|ES*−7.098738552+MUM1|46457|ES*2.479075665	0.928
ME	AMT|64866|ME*−11.67288726+C14orf2|29528|ME*2.439891378+CBWD5|86504|ME*−9.123153517+MEF2A|32717|ME*−5.089591595+FAIM|67014|ME*−3.726223934+CERS5|21668|ME*−2.23329899+STK36|57557|ME*−15.49762863+FBLN5|28892|ME*10.43894503+GLOD4|123198|ME*−4.401329099	0.901
RI	TMUB1|82347|RI*−4.817625749+ELP5|38889|RI*−5.149262596+ZSWIM7|39393|RI*−6.043747899+LRRC29|36982|RI*4.739092712+ZNF276|38138|RI*6.294309106+SNX5|58749|RI*−4.792058193+TTLL3|63208|RI*4.305033369	0.963
All	SEC31A|69730|ES*−3.130605352+RPS24|12295|ES*−2.37334211+FKBP8|48446|AA*−10.11459992+UNG|24277|AP*4.542255311+ARHGEF10|82562|ES*−3.009037952	0.968

Example: “FKBP8|48446|AA*−8.261326216,” “FKBP8” is the gene name, “48446” is the AS id, “AA” is the type of AS event, “FKBP8|48446|AA” mean the PSI value of AA event in FKBP8, and “−8.261326216” is the regression coefficient calculated by the multivariate Cox regression analysis.

### Correlation Network of SF and Survival-Associated AS Events

The data of SFs was obtained from SpliceAid 2 (Piva et al., 2012). SF files and patient survival information were merged and calculated by univariate Cox regression analysis to get survival-associated SFs. Correlation network was constructed using the gene expression of SFs and PSI values of prognosis-related AS events with the conditions of P value less than 0.001 and Pearson correlation coefficient more than 0.7. The correlation network was plotted by Cytoscape (version 3.6.1).

The risk score model based on survival-associated SFs was the sum of each optimal prognostic mRNA expression level multiplying relative regression coefficient weight calculated from the multivariate Cox regression model.

$$\text{Risk Score based on SFs(patient)}=\sum_{i}\text{Coefficient}\left({\text{mRNA}}_{i}\right)\times \text{Expression}\left({\text{mRNA}}_{i}\right)$$

## Results

### Overview of AS Events in pRCC

**Figure 2:**
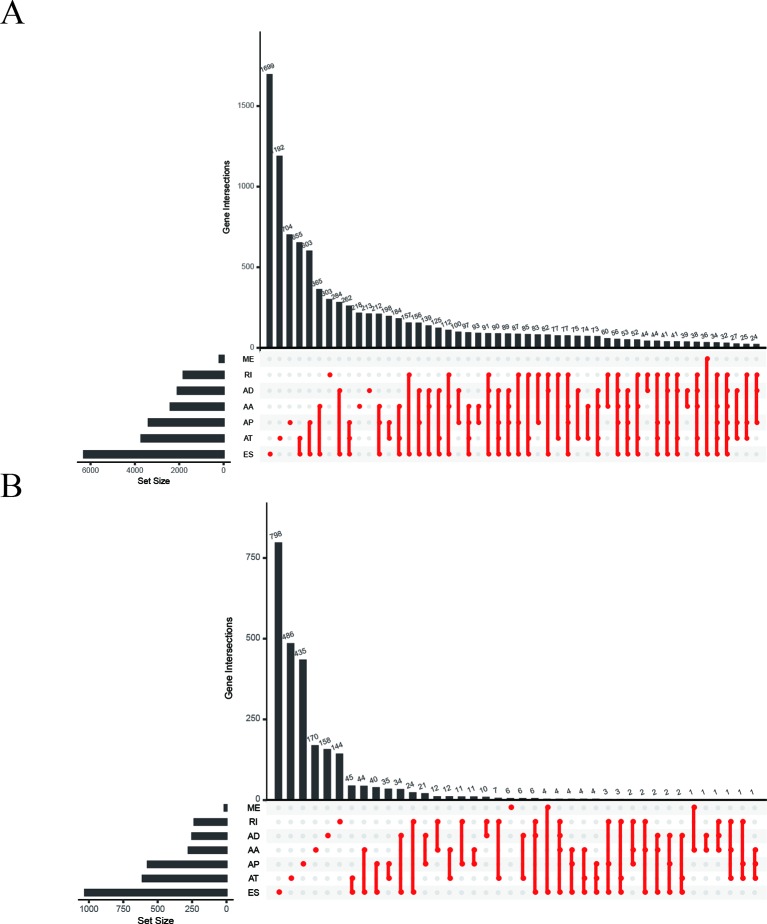
UpSet plots of alternative splicing (AS) events in papillary renal cell carcinoma (pRCC). **(A)** Summary of AS events in pRCC. **(B)** Survival‐associated AS events from univariate Cox regression analysis. AA, alternate acceptor; AD, alternate donor; AP, alternate promoter; AT, alternate terminator; ES, exon skip; ME, mutually exclusive exons; RI, retained intron.

We collected 41,673 AS events from 10,026 genes in 32 adjacent normal tissues and 289 pRCC tissues. The numbers of the genes showing seven types of AS events were plotted by UpSet plot (**Figure 2A**). Several genes only have one kind of AS event, ES was found in 1,699 genes (the largest number) and ME in 36 genes (the smallest number). The plot also showed that one gene might involve two or more AS events, leading to multiple transcripts from one gene. AS data was merged with clinical survival data and calculated by univariate Cox regression analysis. As a result, 4,084 AS events in 2,558 genes were deemed associated with the overall survival (OS) (p < 0.05). The result was also shown by UpSet plot (**Figure 2B**).

### Functional and Pathway Enrichment Analysis

**Figure 3:**
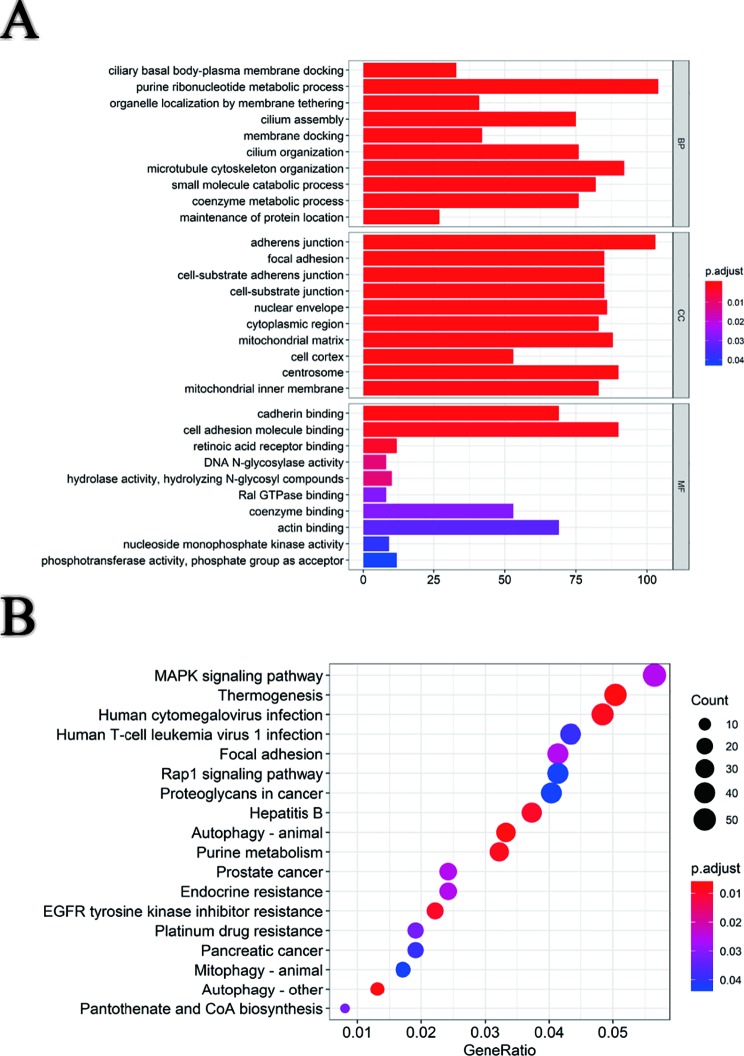
Functional and pathway enrichment analysis. **(A)** Gene ontology analysis of genes with survival-associated alternative splicing events. **(B)** Kyoto Encyclopedia of Genes and Genomes (KEGG) pathway analysis of genes with survival-associated alternative splicing events.

Based on survival-associated AS events, GO (**Figure 3A**), and KEGG (**Figure 3B**) were conducted by “clusterProfiler” package in R. The involved functions and pathways included “ciliary basal body-plasma membrane docking,” “purine ribonucleotide metabolic process,” and “organelle localization by membrane tethering” in biological process (BP), “adherens junction,” “focal adhesion,” and “cell-substrate adherens junction” in cellular component (CC), “cadherin binding,” “cell cadherin molecule binding,” and “retinoic acid receptor binding” in molecular function (MF). Besides, these genes were mainly enriched in “MAPK signaling pathway,” “thermogenesis,” and “human cytomegalovirus infection” in KEGG. AS events generating from these genes might influence the occurrence and development of pRCC through interfering with the above BPs and pathways.

### Prognostic Model for pRCC

**Figure 4:**
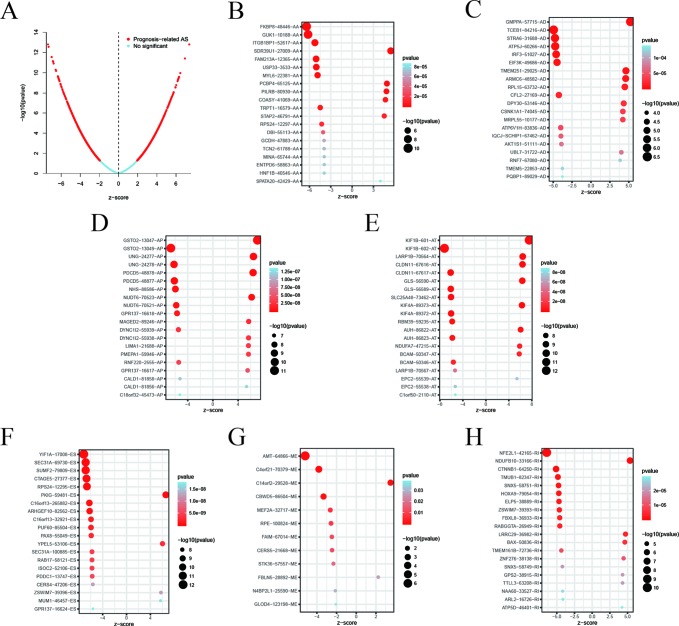
Top 20 most significant alternative splicing (AS) events in papillary renal cell carcinoma (pRCC). **(A)** Volcano plot demonstrating result of univariate Cox regression analysis, red dots represent survival-associated genes and blue dots represent irrelevant genes. The top 20 AS events correlated with clinical outcome based on alternate acceptor (AA) **(B)**, alternate donor (AD) **(C)**, alternate promoter (AP) **(D)**, alternate terminator (AT) **(E)**, exon skip (ES) **(F)**, mutually exclusive exons (ME) **(G)**, and retained intron (RI) **(H)**. The value in the x-axis z-score is the coefficients of univariate cox regression analysis.

**Figure 5:**
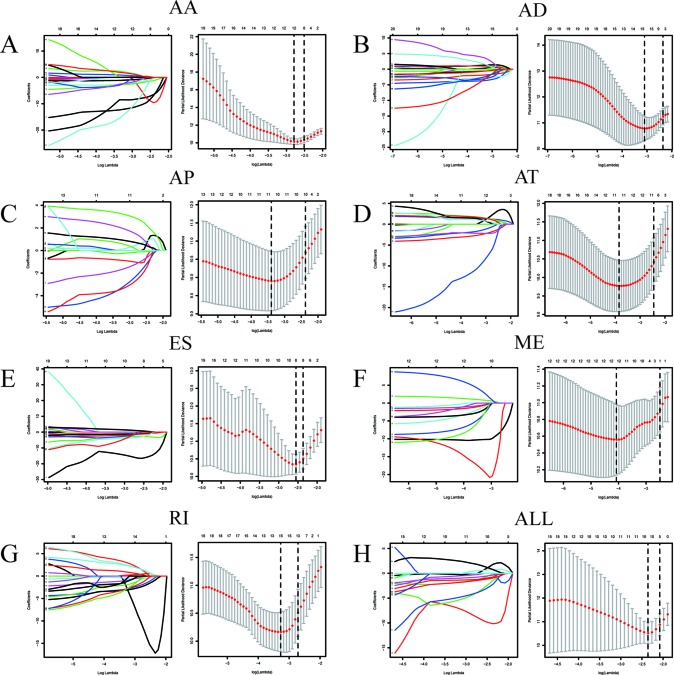
LASSO Cox analysis of alternative splicing (AS) events. LASSO Cox regression model with 10‐fold cross‐validation was constructed using the top significant survival‐associated AS events to screen the key AS features in AA **(A)**, AD **(B)**, AP **(C)**, AT **(D)**, ES **(E)**, ME **(F)**, RI **(G)** and all AS events **(H)**.

**Figure 6:**
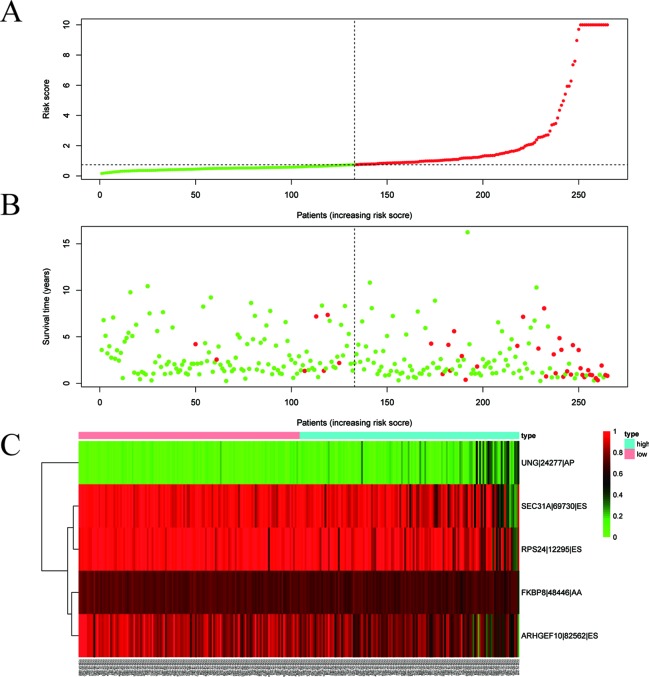
Development of the prognostic index. Risk plot of ALL alternative splicing (AS) event. **(A)** Rank of prognostic index and distribution of groups. Patients with papillary renal cell carcinoma (pRCC) were divided into low- and high-risk subgroups based on the median value of the risk score calculated. **(B)** The survival status and survival time of patients with pRCC ranked by risk score. In **(A)** and **(B)**, green dots represent for patients with a low level of risk score and red dots represent for patients with a high level of risk score. **(C)** Heatmap of included AS event in ALL. Patients were divided into two groups according to risk score. The color from green to red means the Percent Spliced In (PSI) value from 0 to 1.

**Figure 7:**
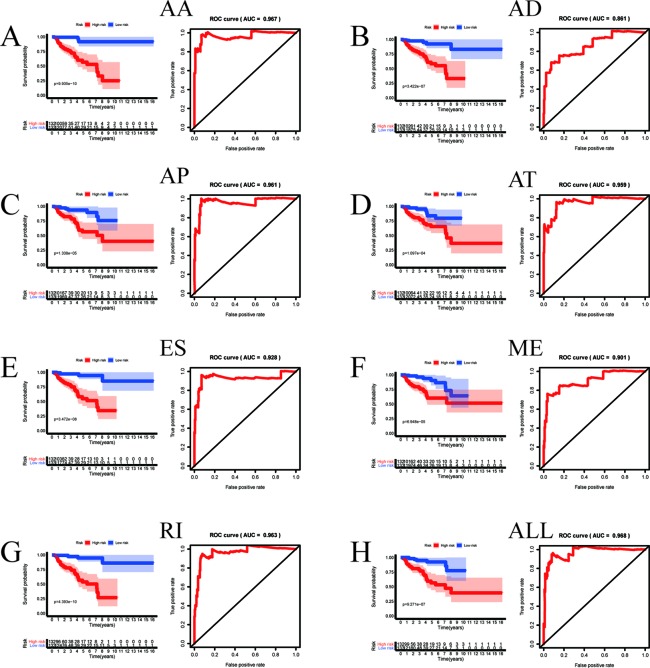
The prognostic value of PI presented by overall survival (OS) and ROC curves. Kaplan-Meier plot depicting the survival probability over time for prognostic predictor of seven types of AS events **(A**–**G)** and all AS event **(H)** with high (red) and low (blue) risk group, respectively. The ROC curve to which the respective model belongs is located to the right of the KM curve.

**Figure 8:**
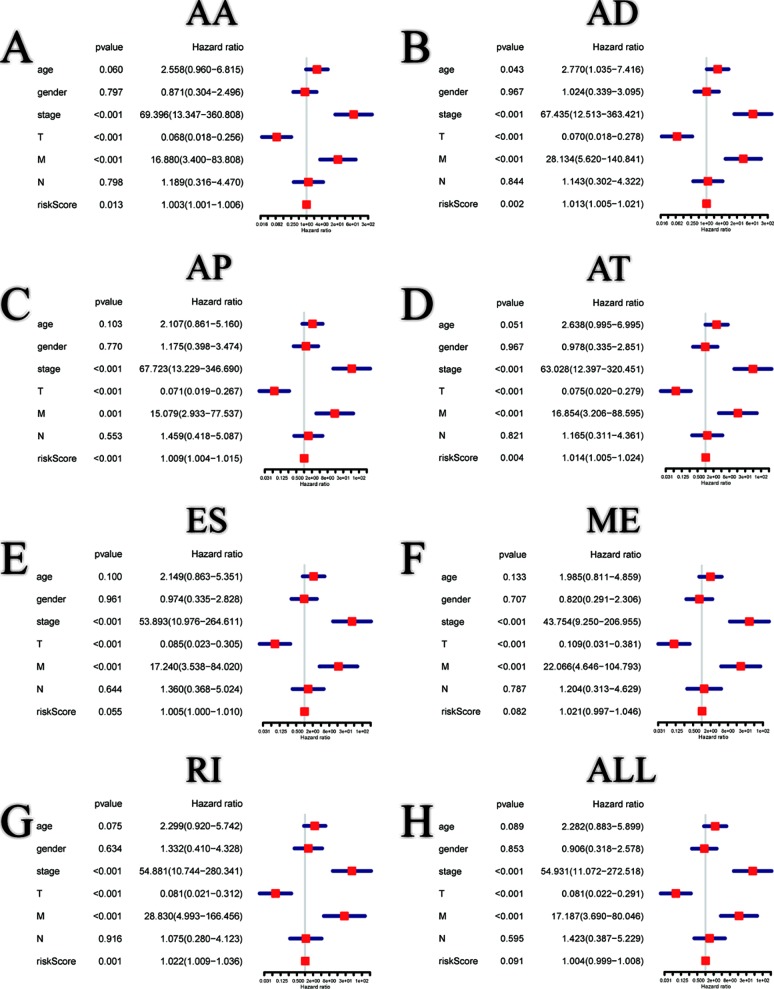
Multivariate Cox regression analysis of clinical parameters and different PI models constructed by AA **(A)**, AD **(B)**, AP **(C)**, AT **(D)**, ES **(E)**, ME **(F)**, RI **(G)** and all AS events **(H)** in pRCC patients.

**Figure 9:**
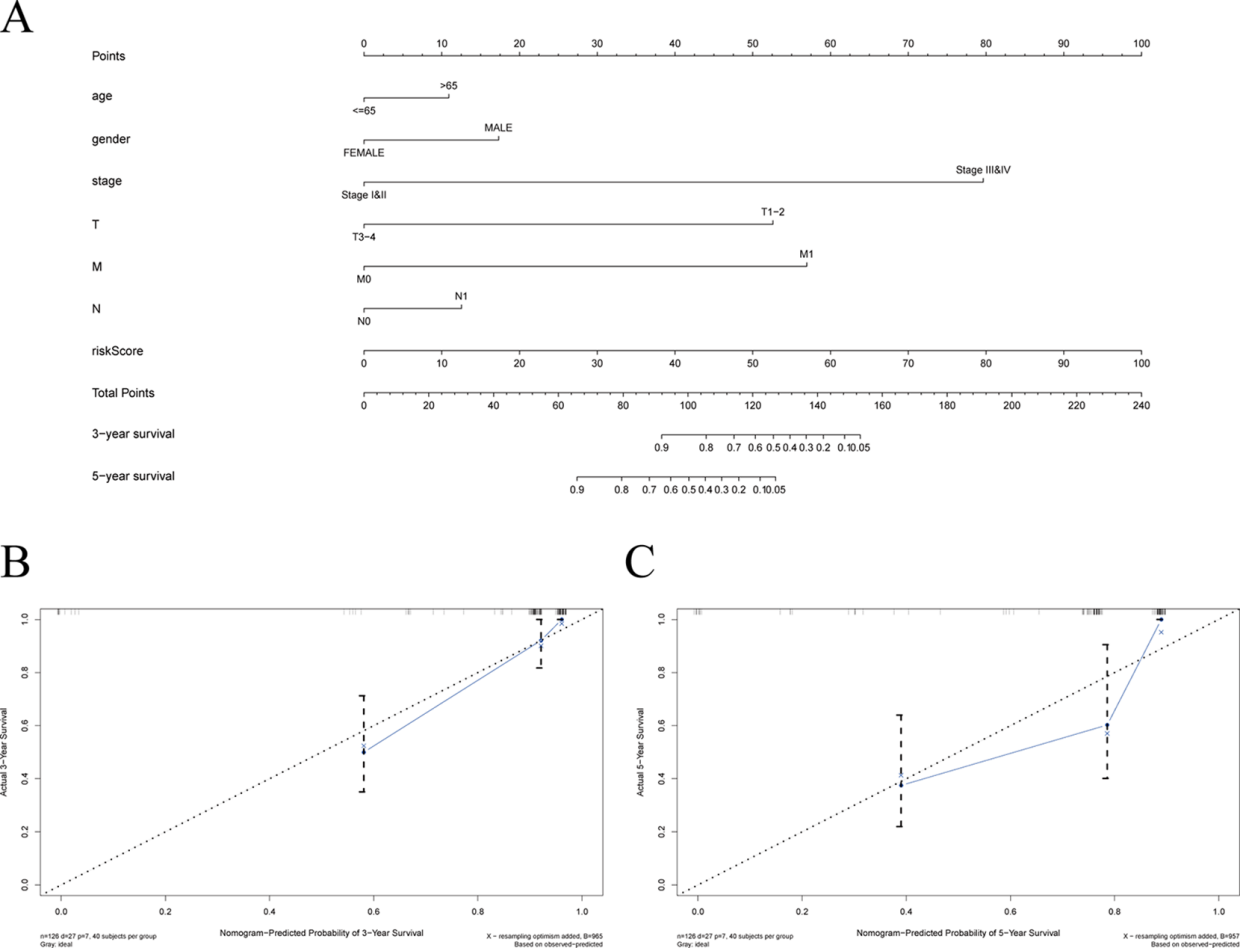
Establishment of the overall survival (OS) nomogram for papillary renal cell carcinoma (pRCC) patients based on alternate acceptor (AA) event. **(A)** Nomogram for predicting OS of pRCC. There were seven factors containing age, gender, stage, T, N, M, and riskscore in the nomogram. Each of them generates points according to the line drawn upward. And the total points of the seven components of an individual patient lie on “Total Points” axis which corresponds to the probability of 3-year and 5-year OS rate plotted on the two axes below. **(B**–**C)** The calibration plots for predicting patient 3‐ or 5‐ year OS.

Among the survival-associated AS events (**Figure 4A**), the top 20 significant AS events were shown by bubble chart (**Figures 4B–H**). These AS events were further treated with LASSO Cox analysis (**Figures 5A–G**). LASSO Cox analysis of all AS events (ALL) was also shown (**Figure 5H**). Next, multivariate Cox analysis was performed to construct an independent model with PI. The formula was showed in **Table 1**. Patients were divided into the low-risk group and the high-risk group based on the median risk score. With the increasing risk score, the patient’s survival became worse. The risk plot of ALL AS events was shown in **Figure 6**. **Figure 6C** with high resolution were affiliated in the **Supplementary Figure 1**. The separate risk plots of seven AS event were affiliated in **Supplementary Figure 2**. The Kaplan-Meier survival analysis suggested that a pRCC patient with a higher risk score might show a worse survival (**Figures 7A–H**). An AUC value of more than 0.9 was found in all the seven types of AS in pRCC except AD (0.861), which validated the efficiency of these signatures in predicting prognosis. To assess whether this model was an independent predictor of pRCC, univariate analyses was performed between clinical factors and risk score. The results showed that this model could distinguish pRCC patients (p < 0.001) (**Table 2**). Furthermore, by using multivariate analyses, this prognostic model was proved to serve as a moderate and independent prognostic indicator in the AS events of AA, AD, AP, AT, and RI. (**Figures 8A–H**). Finally, to provide a clinically associated quantitative method, we tried to construct a nomogram incorporating riskscore and clinical factors to predict the probabilities of 3- and 5-year OS in pRCC. Since the predict model based on AA event showed the best performance among AS events that proved to serve as a moderate and independent prognostic indicator (AUC = 0.967), the nomogram was constructed based on AA event (**Figure 9A**). The Harrel’s concordance index (C-index) for OS prediction was 0.963, which showed a fairly high prediction accuracy of this nomogram. The calibration curves for the 3- (**Figure 9B**) OS rates showed good agreement between the prediction and the actual observation, but not so good in 5-year (**Figure 9C**).

**Table 2:** Univariate analysis between clinical parameters and alternative splicing (AS) events in The Cancer Genome Atlas (TCGA) cohort of papillary renal cell carcinoma (pRCC) patients.

id	HR	HR.95L	HR.95H	pvalue
Age	1.8095854	0.8102357	4.041539	0.1479857
Gender	0.7549775	0.3182836	1.7908277	0.5236151
Stage	12.273306	5.4549378	27.614253	**1.36E-09**
T(Tumor)	5.3163151	2.4726062	11.430533	**1.89E-05**
M(Metastasis)	41.108075	12.143928	139.15381	**2.33E-09**
N (Lymph Node)	12.51509	5.7257522	27.354916	**2.39E-10**
Risk score based on AA	1.0053074	1.0032742	1.0073448	**2.98E-07**
Risk score based on AD	1.0162305	1.0078678	1.0246627	**0.0001341**
Risk score based on AP	1.0088837	1.0050934	1.0126883	**4.12E-06**
Risk score based on AT	1.0223319	1.0147675	1.0299526	**5.58E-09**
Risk score based on ES	1.0106936	1.0065517	1.0148524	**3.84E-07**
Risk score based on ME	1.0523824	1.0325915	1.0725527	**1.36E-07**
Risk score based on RI	1.0257308	1.0171857	1.0343477	**2.65E-09**
Risk score based on ALL	1.0076827	1.0039129	1.0114666	**6.28E-05**

Font bold indicates P < 0.05.

### A Survival-Associated Network Incorporating SFs and AS Events

**Figure 10:**
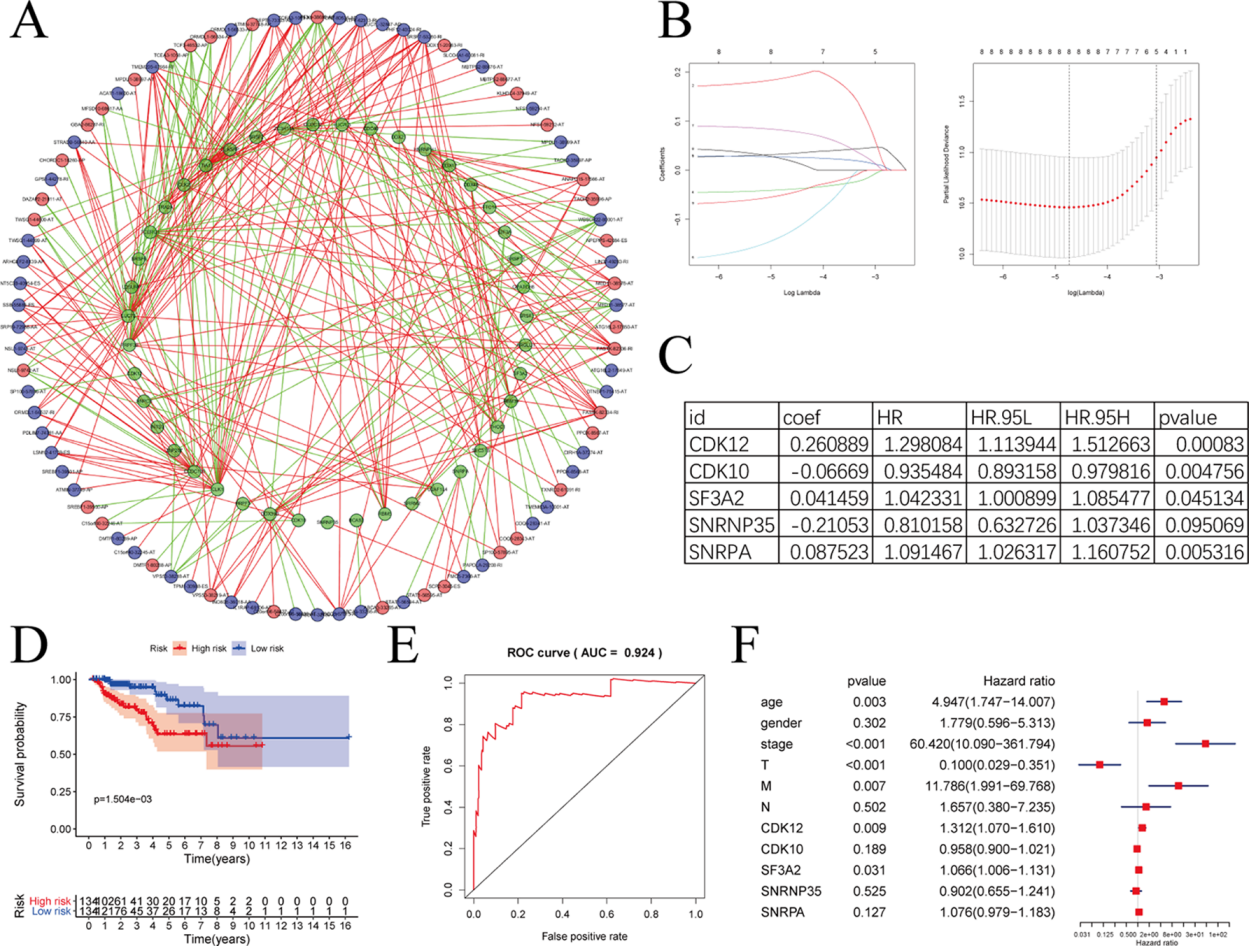
The correlation network between alternative splicing (AS) events and splicing factors in papillary renal cell carcinoma (pRCC). **(A)** Correlation network between filtered AS events and survival-associated splicing factors (SFs). Green dots were survival associated splicing factors. Red/blue dots were favorable/adverse AS events. Red/green lines represent positive/negative correlations between substances. **(B)** LASSO Cox analysis of involved SFs. **(C)** Multivariate Cox regression analysis of LASSO result. **(D**–**E)** The prognostic value of SFs presented by overall survival (OS) and ROC curves. **(F)** Multivariate analyses containing clinical factors and five key genes.

SFs play an important part in the occurrence and development of AS events via changing exon selection and splicing site. Therefore, it is necessary to uncover the correlation between SFs and AS events. Survival analyses based on TCGA data was performed to screen out potential SFs. And then, correlation network was constructed using the expression value of SFs and PSI values of prognosis-related AS events (**Figure 10A**). **Figure 10A** with high resolution were affiliated in the **Supplementary Figure 3**. To highlight the key players in this network, we performed LASSO Cox analysis on those SFs (**Figure 10B**). CDK12, CDK10, SF3A2, SNRNP35, and SNRNA were screened out by multivariate Cox regression analysis (**Figure 10C**).

Risk score = the expression of CDK12 * 0.260889 + the expression of CDK10 * (−0.06669) + the expression of SF3A2 * 0.041459 + the expression of SNRNP35 * (−0.21053) + the expression of SNRPA * 0.087523

The model formed by these five SFs showed outstanding prognostic efficiency as evidenced by the Kaplan-Meier survival curves (**Figure 10D**) and ROC curve (**Figure 10E**). With clinical information, we further analyzed the five key genes with multivariate analyses (**Figure 10F**). In additionally, only CDK12 and SF3A2 were deemed significant statistically (p < 0.05). The HR values of CDK12 and SF3A2 in multivariate analyses were all greater than 1, suggesting that CDK12 and SF3A2 may associate with the poor survival of pRCC patients.

## Discussion

Previous studies have presented that AS is a crucial posttranscriptional regulation leading to structural transcript variation and proteome diversity (Zhang and Manley, 2013). Abnormal AS is associated with tumorigenesis (Grosso et al., 2008). To the best of our knowledge, few systematic AS-related research has been conducted. Prior to our research, Yang XJ et al. identified pRCC into two classes using comparative genomic microarray analysis, one associated with excellent survival and the other with poor prognosis (Yang et al., 2005). Wach S et al. classified pRCC subtypes using microRNA profiles (Wach et al., 2013). Recently, machine learning models have been used to classify stages of PRCC pRCC patients, showing a best performance with area under Precision Recall curve of 0.804, Matthews Correlation Coefficient of 0.711, and accuracy of 88% with Shrunken Centroid classifier on a test dataset based on 80 selected genes (Singh et al., 2018). Therefore, this study is the first systematic analysis on pRCC-survival-associated AS events. The analysis showed that 4,084 AS events in 2,558 genes were associated with the overall survival (OS) of pRCC patients.

The parent genes of survival‐associated AS events were subjected to functional enrichment analyses, and 18 potential pathways were enriched. Among which, MAPK signaling pathway was the top 1 in the list (**Figure 3B**). Targeted therapy against VEGF was a traditional medical treatment for renal cell carcinoma. Zhang Y et al. reported RKTG to inhibit angiogenesis by suppressing MAPK-mediated autocrine VEGF signaling (Zhang et al., 2010), and MAPK signaling pathway has also been reported to be involved in the development of renal cell carcinoma by some other molecular regulation (Huang et al., 2008; Huang et al., 2016; Li et al., 2017). AS is closely related to tumor resistance to drugs (Wang et al., 2017; Martinez-Montiel et al., 2018; Siegfried and Karni, 2018). In the pathway that genes enriched, “EGFR tyrosine kinase inhibitor resistance” and “platium drug resistance” were involved. EGFR (Wei et al., 2013; Robichaux et al., 2018) and platium (Vaughn et al., 2009; Teo et al., 2017; Pal et al., 2018) have been reported to be involved in the treatment of cancer, and through these enriched genes we may be able to discover specific mechanisms of drug resistance.

In this paper, we formed prognostic models by these survival-associated AS events. Before our research, Yang XJ et al performed microarray-based microRNA (miRNA) expression profiling of primary ccRCC and pRCC cases, and finally five miRNAs (miR-145, -200c, -210, -502-3p, and let-7c) were screened out to identify the samples with high accuracy (86.5% in tumor/normal classification, 77.6% in ccRCC/pRCC classification, and 86.4% in pRCC type 1/2 classification); Wach S et al. used machine learning models to classify stages of PRCC pRCC patients, showing a good performance with area under Precision Recall curve of 0.804, Matthews Correlation Coefficient of 0.711 and accuracy of 88% with Shrunken Centroid classifier on a test dataset based on 80 selected genes. As for the prognostic models in our research, firstly, Kaplan-Meier survival curves suggested that these models were appropriate methods to stratify pRCC patients into groups of different survivals (p < 0.01). Secondly, either single AS event or combined seven AS events performed well in predicting overall survival of pRCC patients (AUC > 0.9) with an exception of AUC = 0.861 in AD. Meanwhile, PI was proved to be independent in AA, AD, AP, AT, and RI by univariate and multivariate analyses. Compared with previous studies, using the PSI value of AS events to predict patient’s outcome is theoretically more systematic and accurate. At the same time, by searching for articles, we found that there were also related studies based on AS events in other tumors like uteri corpus endometrial carcinoma (Gao et al., 2019) and papillary thyroid cancer (Lin et al., 2019). However, based on the value of AUC in the prognostic model, it seems that the prognostic model based on AS events is more suitable for pRCC. We also built a nomogram for clinical application and validation.

By searching scientific literature, we found that some genes that make up PI have been reported to play an important role in tumors. For example, in the PI model of AT AS events, CLDN11, SLC25A48, KIF4A, RBM39, BCAM, and LARP1B were involved (**Table 1**, AT). It was reported that inactivation of CLDN11 could promote cell migration in nasopharyngeal carcinoma (Li et al., 2018). KIF4A were identified as prognostic gene or key gene involved in the metastasis of renal cell carcinoma in recent survey (Gu et al., 2017; Wei et al., 2019). Anticancer sulfonamides functions by inducing RBM39 degradation (Anticancer Sulfonamides Induce Splicing Factor RBM39 Degradation, 2017; Han et al., 2017). BCAM was reported to mediate recognition between tumor cells and the endothelium in KRAS-Mutant colorectal cancer. In the present study, the role of SLC25A48 and LARP1B was still unclear, and our analysis may guide the direction of future research on pRCC.

SFs are implicated in the process of alternative mRNA splicing (Venables et al., 2009). Splicing abnormalities arise owing to aberrant expression and/or mutations of SFs (Venables, 2006). Hence, SFs have a tight link with AS events. In this paper, survival-associated SFs were screened out by survival analyses. Correlation network was then formed to describe the interactions between SFs and AS events. Both positive and negative correlations were observed between one SF and multiple survival-associated AS events, or between one survival-associated AS event and multiple SFs. By performing LASSO Cox analysis and multivariate Cox regression analysis, CDK12, CDK10, SF3A2, and SNRNA were screened out. However, only CDK12 and SF3A2 were deemed significant statistically (p < 0.05) according to multivariate analyses. CDK12 contains an arginine–serine-rich (RS) domain, and can regulate the splicing of a minigene construct (Chen et al., 2006). CDK12 may be inactivated in patients with metastatic castration-resistant prostate cancer, and may make tumors more responsive to PD-1 inhibitors (CDK12 Changes Telling in Prostate Cancer, 2018). According to the regulatory network in our research, we found a negative correlation between CDK12 and SP100-57896-AT. It has been reported that SP100 could reduce malignancy of human glioma cells (Held-Feindt et al., 2011). The HR value of SF3A2 was 1.051, and SF3A2 was also reported to be associated with the metastasis and recurrence of osteosarcoma (Zhang et al., 2019). These results indicated that these altered SFs, as independent molecules, can construct a regulatory network in the carcinogenesis and progression in pRCC. However, this network may be optimized with more molecules. Besides, only 129 pRCC patients were involved in our analysis due to the restricted standard of OS time more than 90 days and requirement for complete clinical data. PI was proved to be independent in AA, AD, AP, AT, and RI by univariate and multivariate analyses in this paper, but when we brought all the AS events together, p > 0.05 in multivariate analyses (**Figure 8H**) which indicted that there were still some key factors that were not considered in our analysis and certain errors were inevitable due to the heterogeneity of patients.

In conclusion, our study created an efficient prognostic model based on survival-associated AS events for pRCC, which may help clinicians in selecting reliable prognostic indicators and understanding the mechanism of pRCC.

## Data Availability Statement

Publicly available datasets were analyzed in this study. This data can be found here: https://portal.gdc.cancer.gov/repository. AS files could be downloaded from TCGASpliceSeq: https://bioinformatics.mdanderson.org/TCGASpliceSeq/.

## Author Contributions

Conception and design: XX and CC. Collection and assembly of data: ZW and JL. Data analysis and interpretation: RS and DC. Manuscript writing: ZW and KW. Final approval of manuscript: All authors.

## Conflict of Interest

The authors declare that the research was conducted in the absence of any commercial or financial relationships that could be construed as a potential conflict of interest.
