# Supplementary figures and images for: A Novel Prognostic Index Based on Alternative Splicing in Papillary Renal Cell Carcinoma
Source: Front Genet. 2020 Jan 29;10:1333. doi: 10.3389/fgene.2019.01333 (PMC6999693; doi:10.3389/fgene.2019.01333)

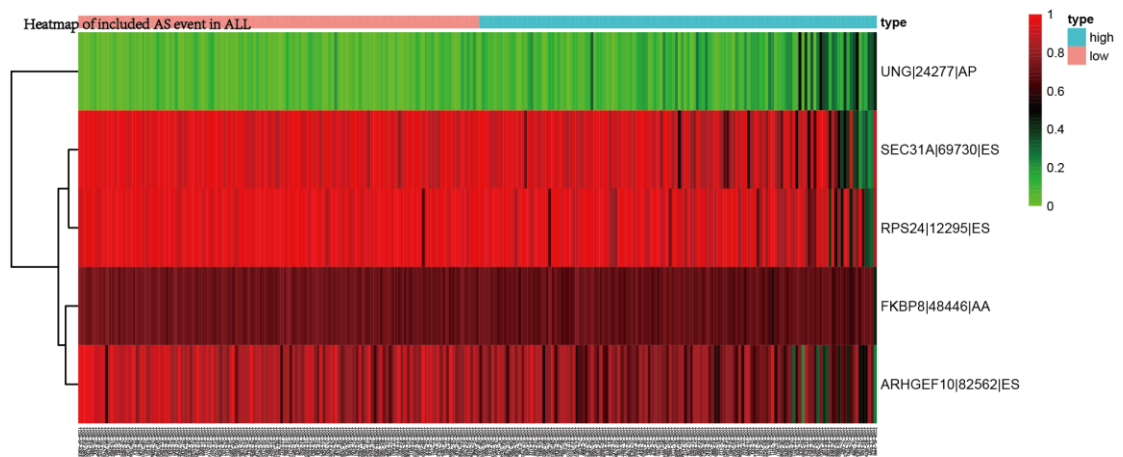

**Supplementary image 1** | Figure 6C with high-resolution

Supplement: Supplementary file 1 [file Image_1.pdf]

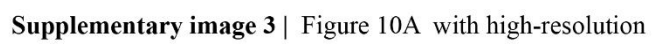

**Supplementary image 3** | Figure 10A with high-resolution

Supplement: Supplementary file 3 [file Image_3.pdf]
