# Supplementary material for: A Novel Prognostic Index Based on Alternative Splicing in Papillary Renal Cell Carcinoma
Source: Front Genet. 2020 Jan 29;10:1333. doi: 10.3389/fgene.2019.01333 (PMC6999693; doi:10.3389/fgene.2019.01333)
Supplement: Supplementary file 2 [file Image_2.pdf]

Risk plot of seven type AS event

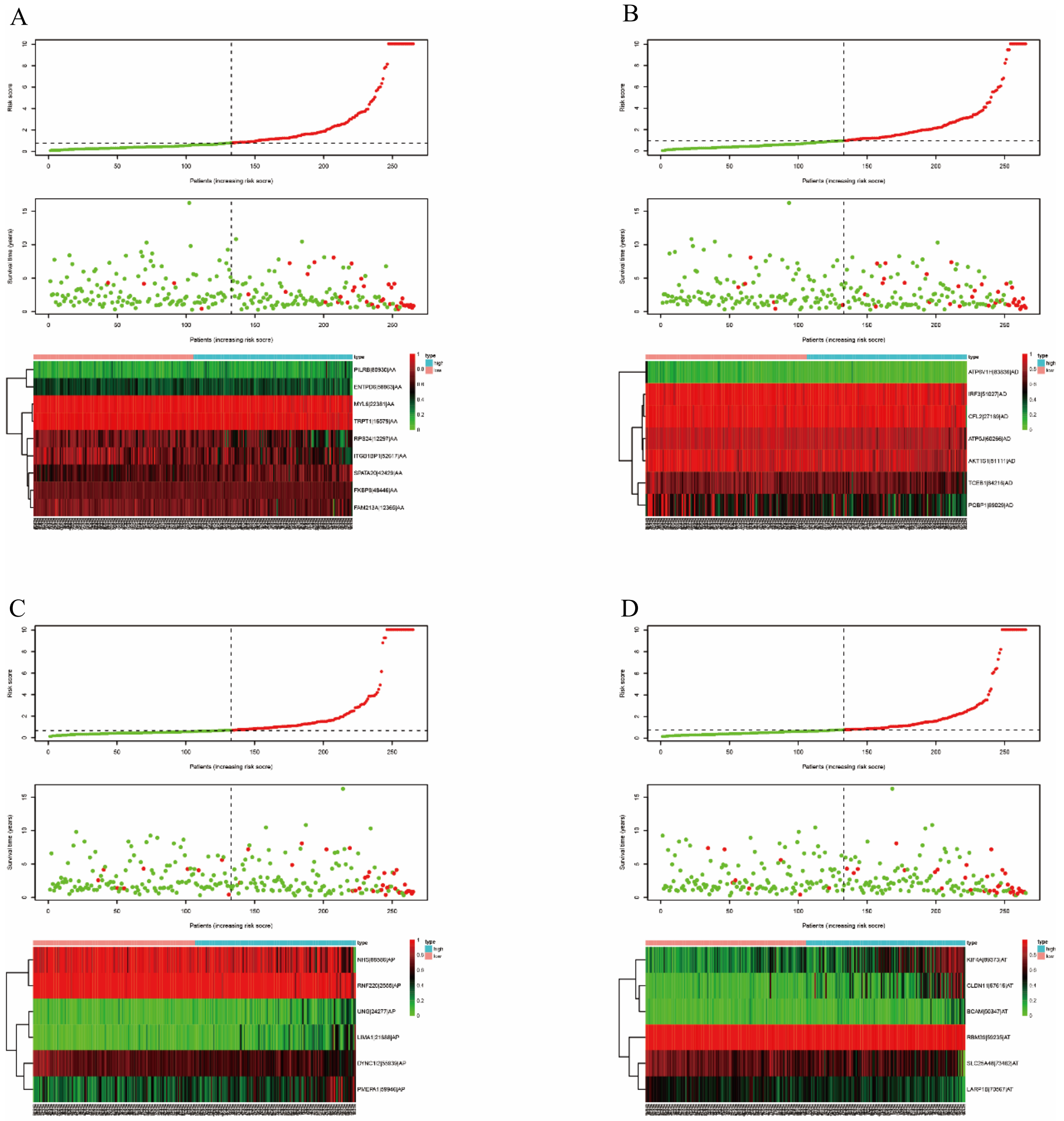

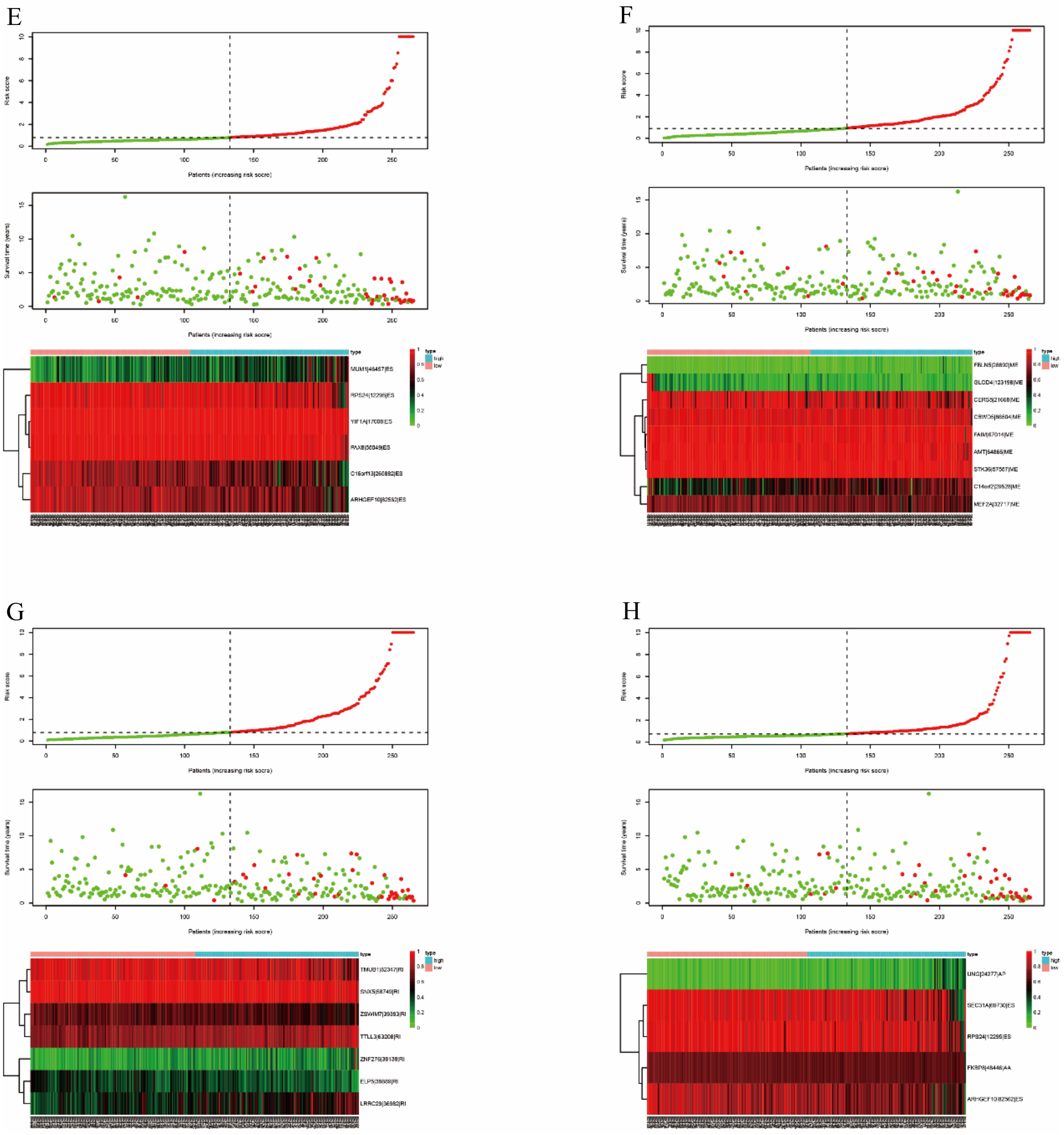

Supplementary image 2 | The risk plot of AS event in AA (A), AD (B), AP(C), AT(D), ES(E), ME(F), RI (G) and in ALL (H).
